# Invasive Meningococcal X Disease during the COVID-19 Pandemic, Brazil

**DOI:** 10.3201/eid2809.220531

**Published:** 2022-09

**Authors:** Lucila O. Fukasawa, Bernadete L. Liphaus, Maria Gisele Gonçalves, Fabio T. Higa, Carlos H. Camargo, Telma R.M.P. Carvalhanas, Ana Paula S. Lemos

**Affiliations:** Adolfo Lutz Institute, São Paulo, Brazil (L.O. Fukasawa, M.G. Gonçalves, F.T. Higa, C.H. Camargo, A.P.S. Lemos);; Epidemiological Surveillance Center “Professor Alexandre Vranjac,” São Paulo (B.L. Liphaus, T.R.M.P. Carvalhanas)

**Keywords:** Meningococcal X disease, COVID-19, Brazil, SARs-CoV-2, meningitis/encephalitis, *Neisseira meningitidis* serogroup X, MenX, invasive meningococcal disease

## Abstract

Invasive meningococcal disease persists as a fulminant disorder worldwide. Although cases caused by *Neisseria meningitidis* serogroup X (MenX) occur infrequently, outbreaks have been reported in countries in Africa in recent decades. We report 2 cases of MenX invasive meningococcal disease in São Paulo, Brazil, in 2021 and 2022, during the COVID-19 pandemic.

Invasive meningococcal disease (IMD) is a severe disorder that is associated with high rates of morbidity and mortality worldwide (*1*). Although 12 serogroups of *Neisseria meningitidis* have been characterized based on their capsular polysaccharides, most IMD cases are caused by serogroups A, B, C, W, Y, and, more rarely, X (*1*).

*N. meningitidis* serogroup X (MenX) has been responsible for limited IMD cases in the United States and Europe, but since 1990, MenX isolates have emerged in some countries within the meningitis belt of Africa, causing outbreaks and epidemics in Burkina Faso, Togo, Niger, Kenya, and Uganda (*2*). In Brazil, only 6 cases of MenX IMD cases were reported in the last 15 years; the last one was isolated in the city of São Paulo in 2017 (http://tabnet.datasus.gov.br/cgi/tabcgi.exe?sinannet/cnv/meninbr.def). We report 2 cases of MenX IMD that occurred in São Paulo in 2021 and 2022, during the COVID-19 pandemic. These isolates were identified during routine laboratory-based public health surveillance in the National Reference Laboratory at the Adolfo Lutz Institute in São Paulo.

Case-patient 1 was a 7-month-old boy who, in November 2021, was admitted to a São Paulo emergency department with fever, vomiting, bulging anterior fontanelle, stiff neck, and seizure. Cerebrospinal fluid collected at that time revealed a leukocyte count of 1,440 cells/mm^3^ with a 73% proportion of neutrophils; protein level was 263 mg/dL, glucose 19 mg/dL, and lactate 80.5 mg/dL, and results of bacterioscopy and culture were negative. The patient was treated with ceftriaxone (100 mg/kg every 12 h) for 10 days, with a favorable outcome.

Case-patient 2 was a 6-year old boy who, in January 2022, was admitted to a São Paulo City emergency department with fever, headache, and vomiting. A sample of cerebrospinal fluid revealed a leukocyte count of 4920/mm^3^ with a 96% proportion of neutrophils; protein level was 207 mg/dL, glucose 48 mg/dL, and lactate 82.3 mg/dL, and results for bacterioscopy and culture were negative. The patient was treated with ceftriaxone (100 mg/kg every 12 h), the recommended antibiotic, with a favorable outcome.

Because both patients were diagnosed with meningitis, chemoprophylaxis with rifampin was administered to all persons characterized as close contacts. Both patients resided in the city of São Paulo but in different regions, 40 km away from each other. Despite the short period between their illnesses, epidemiologic surveillance could not establish an obvious relationship between the 2 patients. Neither patient had traveled to countries with reported MenX disease, nor had they had known contact with other persons diagnosed with meningitis.

We extracted DNA from the cerebrospinal fluid samples obtained from the 2 patients using the Roche MagNa Pure LC 2.0 platform (https://lifescience.roche.com) according to the manufacturer’s instructions. Both DNA samples were positive for *N. meningitidis* (*ctrA* gene) by multiplex real-time PCR (*3*), and these results were confirmed using another real-time PCR targeting the meningococcal *sodC* gene (*4*). Both samples were positive for genogroup X (*xcbB* gene) by multiplex real-time PCR and negative for genogroups A, B, C, W, and Y (*5*).

A previous study demonstrated that the 413 bp fragment of the *rplF* gene, which encodes the 50S ribosomal protein L6, is a suitable genetic target for differentiating species within the genus *Neisseria* (*6*). A sequence analysis of the *rplF* gene of both of our patient samples revealed an *rplF* fragment assigned to allele 1, confirming the genospecies *N. meningitidis* (*6*). We conducted multilocus sequence typing according to standard protocols using the the PubMLST database (https://pubmlst.org/neisseria) and identified Nm400, the isolate from case-patient 2, as sequence type 2888, which is not assigned to a clonal complex. Because of the unavailability of clinical material for Nm111, the isolate from case-patient 1, multilocus sequence typing could not be performed for that isolate. 

According to the PubMLST database (as of March 12, 2022), there are 4 records of ST2888: 1 is serogroup X, 2 are serogroup B, and 1 isolate did not have a serogroup recorded. Only 1 case of IMD caused by MenX ST2888 was reported, in Italy in 2009, in a patient who had not traveled abroad and who had a favorable outcome (*7*).

A large study in sub-Saharan Africa showed that the current population structure reveals MenX to be of a predominantly single lineage—ST181 of MenX belongs to a single main lineage, ST181 (clonal complex 181)—which is unlike the diversified MenX population found in Europe (*8*). A recent study in Italy described 4 cases of serogroup X IMD among refugees: 3 were immigrants from Africa, and the other was an immigrant from Bangladesh who had been in contact with refugees from Africa for several months. This highlights the potential threat of new lineages being introduced into vulnerable populations in times of humanitarian crises and conflicts (*9*).

Given the COVID-19 pandemic scenario that has brought an interruption in nonpharmacologic measures to control SARS-CoV-2 transmission, and considering the globally observed reduction of meningococcal vaccination coverage (*10*), a resurgence in cases of IMD will be likely, and cases of non–vaccine-preventable serogroup X should be monitored. Ongoing surveillance of IMD, as well as addition of surveillance initiatives in some regions, will be needed to ensure a successful public health response in terms of both prevention and control.
